# [Corrigendum] Umbilical cord-derived mesenchymal stem cells promote proliferation and migration in MCF-7 and MDA-MB-231 breast cancer cells through activation of the ERK pathway

**DOI:** 10.3892/or.2025.8933

**Published:** 2025-06-18

**Authors:** Tao Li, Chunfu Zhang, Yanling Ding, Wei Zhai, Kui Liu, Fan Bu, Tao Tu, Lingxian Sun, Wei Zhu, Fangfang Zhou, Wenkai Qi, Jiabo Hu, Huabiao Chen, Xiaochun Sun

Oncol Rep 34: 1469–1477, 2015; DOI: 10.3892/or.2015.4109

Subsequently to the publication of the above paper, an interested reader drew to the authors' attention that a pair of data panels appeared to contain overlapping data in each of Fig. 3A and C on p. 1473, showing the results of Transwell migration and scratch-wound assay experiments respectively, where the data panels were intended to have shown the results from differently performed experiments; moreover, control GAPDH western blotting data featured in Fig. 4B and 5A appeared to be matching, albeit the bands had been inserted into these figure parts in reversed orientations.

The authors were able to re-examine their original data, and realized that errors had occurred in the assembly of these figures through mislabelling of certain of their data files. Owing to the time that had passed since this paper was published, the authors offered to repeat the affected experiments, and the revised versions of Figs. 3, 4 and 5, showing new data for Figs. 3A, 3C, 4B and 5A, are shown on the next two pages. Given that the new experiments yielded data that were not significantly different from those obtained previously, the authors wish to emphasize that the re-presentation of these figures with the new data does not affect the overall conclusions reported in the paper. The authors are grateful to the Editor of *Oncology Reports* for allowing them the opportunity to publish this corrigendum, and all the authors agree with the publication of this corrigendum; moreover, they apologize to the readership for any inconvenience caused.

## Figures and Tables

**Figure 3.**
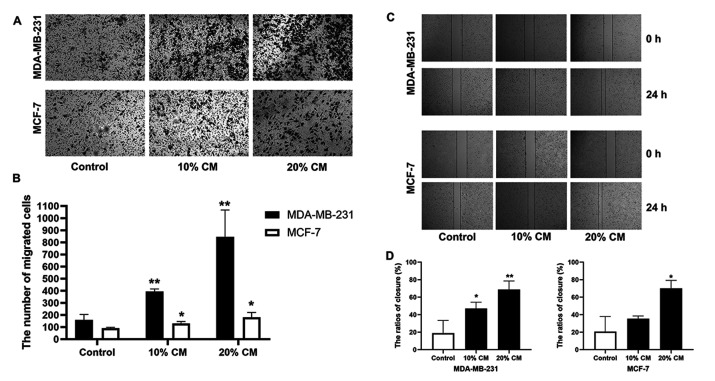
UC-MSCs promote the migration of MCF-7 and MDA-MB-231 cells. (A) Results of the Transwell migration assay of MCF-7 and MDA-MB-231 cells. (B) Number of migrated cells in the different groups. There were significant differences between the 10 and 20% CM groups and the control groups in terms of the number of migrated cells. Magnification, ×200. (C) Wound-healing assay results. Example images of wound closure in the control, 10 and 20% CM groups. (D) Wound closure ratios in the different groups. There were significant differences between the 10 and 20% CM groups and the control groups. *p<0.05 and **p<0.01 compared with the control group. Magnification, ×100. CM, conditioned medium; UC-MSCs, umbilical cord mesenchymal stem cells..

**Figure 4.**
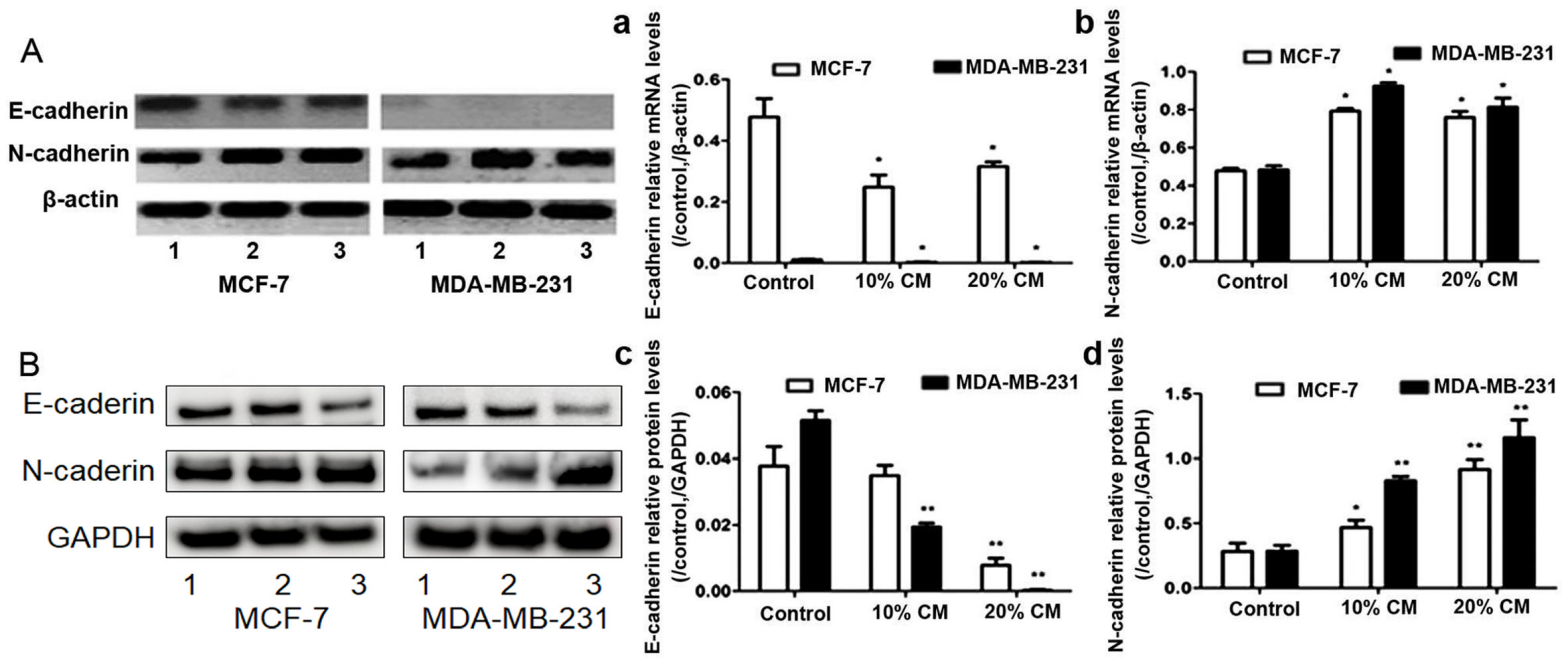
E-cadherin and N-cadherin expression. (A) The E-cadherin and N-cadherin mRNA levels were analyzed by RT-PCR, and β-actin mRNA was used as a control to ensure equal loading. Three independent experiments for measuring the (a) E-cadherin and (b) N-cadherin mRNA levels. (B) The E-cadherin and N-cadherin protein levels in MCF-7 and MDA-MB-231 were analyzed by western blotting. GAPDH protein was used as a control to ensure equal loading. Three independent experiments for measuring the (c) E-cadherin and (d) N-cadherin protein levels. *p<0.05 and **p<0.01 compared with the control group. Lane 1, control; lane 2, 10% CM; lane 3, 20% CM. CM, conditioned medium.

**Figure 5.**
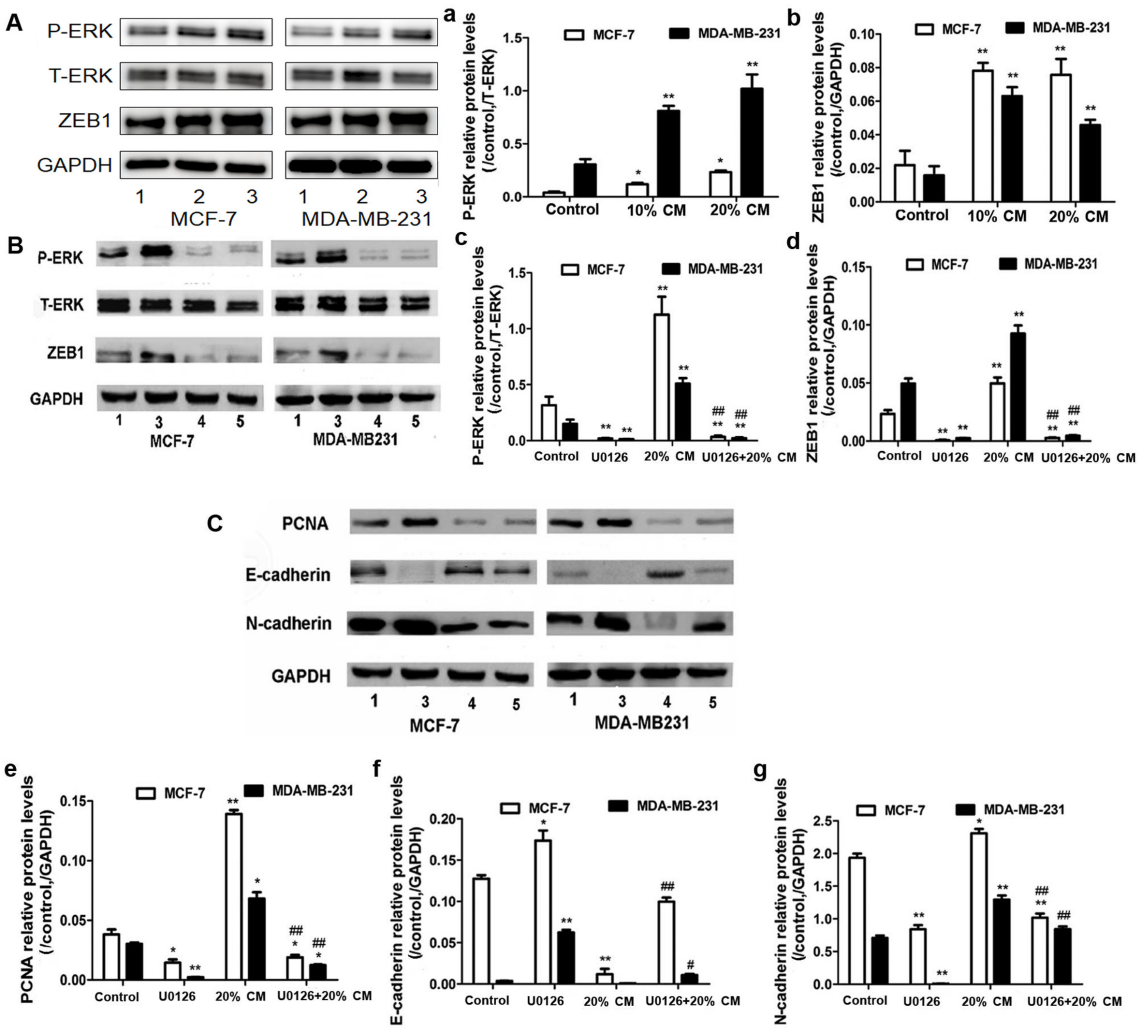
Effects of UC-MSCs and ERK inhibitor U0126 on the protein expression levels in the breast cancer cell lines. (A) MCF-7 and MDA-MB-231 cells were treated with 0, 10 and 20% UC-MSC-CM for 48 h. The P-ERK and ZEB1 protein levels in MCF-7 and MDA-MB-231 cells were analyzed by western blotting. T-ERK and GAPDH protein levels were used as controls to ensure equal loading. Three independent experiments were performed to measure the (a) P-ERK and (b) ZEB1 protein levels. (B and C) MCF-7 and MDA-MB-231 cells were pretreated with 10 µM U0126 for 60 min before the addition of 0 and 20% UC-MSC-CM. The P-ERK, ZEB1, E-cadherin, N-cadherin, and PCNA protein levels were analyzed by western blotting. Three independent experiments were used to measure the (c) P-ERK, (d) ZEB1, (e) PCNA, (f) E-cadherin and (g) N-cadherin protein levels. *p<0.05 and **p<0.01 compared with the control group; ^#^p<0.05 and ^##^p<0.01 for the comparison between the U0126+20% CM group and the 20% CM group. Lane 1, control; lane 2, 10% CM; lane 3, 20% CM; lane 4, U0126; lane 5, U0126+20% CM. CM, conditioned medium; UC-MSCs, umbilical cord mesenchymal stem cells.

